# Multimodal Therapeutic Effects of Neural Precursor Cells Derived from Human-Induced Pluripotent Stem Cells through Episomal Plasmid-Based Reprogramming in a Rodent Model of Ischemic Stroke

**DOI:** 10.1155/2020/4061516

**Published:** 2020-03-23

**Authors:** Seung-Hun Oh, Yong-Woo Jeong, Wankyu Choi, Jeong-Eun Noh, Suji Lee, Hyun-Sook Kim, Jihwan Song

**Affiliations:** ^1^Department of Neurology, CHA Bundang Medical Center, CHA University, Seongnam, Gyeonggi-do, Republic of Korea; ^2^CHA Stem Cell Institute, Department of Biomedical Science, CHA University, Seongnam, Gyeonggi-do, Republic of Korea; ^3^iPS Bio, Inc., Seongnam, Gyeonggi-do, Republic of Korea

## Abstract

Stem cell therapy is a promising option for treating functional deficits in the stroke-damaged brain. Induced pluripotent stem cells (iPSCs) are attractive sources for cell therapy as they can be efficiently differentiated into neural lineages. Episomal plasmids (EPs) containing reprogramming factors can induce nonviral, integration-free iPSCs. Thus, iPSCs generated by an EP-based reprogramming technique (ep-iPSCs) have an advantage over gene-integrating iPSCs for clinical applications. However, there are few studies regarding the *in vivo* efficacy of ep-iPSCs. In this study, we investigated the therapeutic potential of intracerebral transplantation of neural precursor cells differentiated from ep-iPSCs (ep-iPSC-NPCs) in a rodent stroke model. The ep-iPSC-NPCs were transplanted intracerebrally in a peri-infarct area in a rodent stroke model. Rats transplanted with fibroblasts and vehicle were used as controls. The ep-iPSC-NPC-transplanted animals exhibited functional improvements in behavioral and electrophysiological tests. A small proportion of ep-iPSC-NPCs were detected up to 12 weeks after transplantation and were differentiated into both neuronal and glial lineages. In addition, transplanted cells promoted endogenous brain repair, presumably via increased subventricular zone neurogenesis, and reduced poststroke inflammation and glial scar formation. Taken together, these results strongly suggest that intracerebral transplantation of ep-iPSC-NPCs is a useful therapeutic option to treat clinical stroke through multimodal therapeutic mechanisms.

## 1. Introduction

Worldwide, stroke is one of the most serious brain disorders [1]. Although some patients show spontaneous recovery after stroke, more than 30% of patients have permanent functional deficits despite intensive efforts of rehabilitation [2]. Residual deficits following stroke present serious economic and mental problems for patients and their families. To date, treatment of ischemic stroke depends on the use of tissue-type plasminogen activator, a thrombolytic agent that works only within 4.5 h after the onset of stroke [3]. However, there is currently no established treatment for the chronic phase of stroke. Restoration of neurons in the damaged brain is a prerequisite for functional improvement in patients with chronic stroke.

Stem cell therapy is one of the most attractive targets for the treatment of chronic stroke [4]. To date, different types of stem cells have been investigated for cell therapy in stroke [5]. The most widely used cells in stroke research are mesenchymal stem cells (MSCs) for reasons of easier access from tissues and reduced ethical concerns. MSCs exert their therapeutic effects via the paracrine activity of their secreted trophic factors [6]. However, evidence for the differentiating capacity of MSCs into functional neurons does not exist. In contrast, embryonic stem cells (ESCs) show infinite self-renewal and the ability to differentiate into almost any cell type in the body. However, ESCs have several problems in their clinical application, such as ethical problems, the allogeneic origin of cells, and the induction of tumorigenesis.

Induced pluripotent stem cells (iPSCs) provide a therapeutic opportunity for the use of patient-specific somatic cells in many diseases. iPSCs have self-renewal and differentiation potentials similar to ESCs [7]. However, iPSCs have greater advantages compared to ESCs because the former can be generated from the patient's own somatic cells and therefore avoid immune rejection when transplanted. In addition, ethical problems do not surround their use and invasive surgery is not required to obtain cells. iPSCs can be generated from different types of somatic cells, including skin fibroblasts, keratinocytes, or peripheral blood [8]. Of these sources, peripheral blood mononuclear cells (PBMCs) have some advantages over other tissues for iPSC generation because they are easily obtained without the need for invasive surgical procedures [8–10]. Several *in vivo* studies of iPSC transplantation in animal stroke models demonstrated an improvement in neurological functions following stroke [11–21]. These results highlight iPSCs as a promising therapeutic option for stroke. However, iPSCs show several limitations for cell therapy. For example, in the case of iPSCs generated by retroviral-based gene delivery, exogenous DNA in viral vectors can integrate into the endogenous genomes of cells [7]. Therefore, several nonviral, integration-free methods have been investigated to overcome such issues. The use of episomal plasmids (EPs) for delivery of reprogramming factors is one of the options in nonviral gene delivery for the generation of iPSCs [10, 22, 23]. This EP-based reprogramming technology is a unique alternative to traditional retroviral-based reprogramming of somatic cells for iPSC generation. However, there have been few preclinical studies of iPSC transplantation in an animal stroke model using integration-free methods [21, 24]. Here, we investigated the therapeutic potential of the transplantation of neural precursor cells derived from iPSCs using an EP-based reprogramming technique (ep-iPSC-NPCs) in a rodent stroke model.

## 2. Materials and Methods

### 2.1. Ethics Statement

This study was conducted in accordance with the CHA University Institutional Animal Care and Use Committee on animal experiments (IACUC, approval no. 150066). The present study followed ARRIVE guidelines for the reporting of animal research. Animals were housed in standard laboratory cages (12 h light/dark cycles, temperature 23 ± 1°C), two in each cage, with access to food and water *ad libitum*.

### 2.2. Preparation of ep-iPSC-Derived Neural Precursor Cells

The human iPSC line generated from a human PBMC (1231A3) was provided by the Center for iPS Cell Research and Application (CiRA) at Kyoto University, Japan. The 1231A3 iPSC line was originally generated by the electroporation of EP vectors consisting of OCT3/4, KLF4, SOX2, L-Myc, Lin28, p53 dominant-negative mutant (mp53DD), and Epstein–Barr nuclear antigen 1 (EBNA1). Detailed methods for the generation of ep-iPSCs have been described elsewhere [8, 23]. The ep-iPSCs were maintained under feeder-free and xeno-free conditions using the StemFit™ (Ajinomoto, Tokyo, Japan) medium and cultured on 35 mm tissue culture dishes (BD, Franklin Lakes, NJ, USA; 353002) coated with 0.5 mg/cm^2^ iMatrix-511 (Matrixome, Osaka, Japan). Also, tests for pluripotency marker expression, karyotype stability, and three-germ layer formation were performed to validate the characteristics of the ep-iPSCs.

When preparing cells for transplantation, ep-iPSCs were induced into neural precursor cells (NPCs) with the use of small molecules, including dual SMAD inhibitors, such as SB-431542 and LDN-139189 (see below). To differentiate cells, we cultured ep-iPSCs in initial differentiation medium conditions containing Dulbecco's Modified Eagle Medium (DMEM)/F12, 5% knock-out serum replacement (KOSR), 10 *μ*M SB-431542 (Reagent Direct, Encinitas, CA, USA; 21-A94), and 100 nM LDN-139189 (Reagent Direct; 36-F52) for 3 days. We then changed this to a neural induction medium to include DMEM/F12 with an N2 supplement (Gibco, Waltham, MA, USA; 17502-048), 1 mM sodium pyruvate (Gibco; 11360-070), 3 mM D-glucose (Gibco; G7021), 2 mM L-glutamine (Welgene, Daegu, Korea; LS002-01), 0.2 mM ascorbic acid (Sigma, St. Louis, MI, USA; A4544), and 3 *μ*M CHIR 99021 (Tocris, Bristol, UK; 4423). The medium was changed every day for 2 days. After that, when we observed rosette-like structures; cells were carefully dissociated using Accutase (Stem Cell Technologies, Vancouver, Canada; 07920) at 37°C, in 5% CO_2_ for 10 min, and transferred to a tissue culture dish coated with 20 *μ*g/mL poly-L-ornithine (PLO) and 5 *μ*g/mL laminin (LN). At this stage, we used a neural induction medium containing N2/B27 (Gibco; 17504-044) and basic fibroblast growth factor (Peprotech, Rocky Hill, NJ, USA; 100-18B). For transplantation, ep-iPSC-NPCs were carefully dissociated using a 0.5x concentration of TrypLE Select (Gibco; 12563-011) in 0.5 mM EDTA (Gibco; 15575-020).

### 2.3. Rodent Stroke Model

A stroke model was induced by the middle cerebral artery occlusion (MCAo) for 90 min [25]. Adult male Sprague–Dawley rats (Orients, Seoul, Korea) weighing 270 g to 300 g were used in these experiments. After anesthesia with 1% ketamine (57.6 mg/kg, intraperitoneally (i.p.)) and xylazine (7.7 mg/kg, i.p.), rats were maintained at a body temperature of 37 ± 1°C by lying on a heating pad. The carotid artery was exposed, and a blunt-ended silicon coating monofilament (Ethicon, Pinewood, UK; 4-0) was inserted to occlude the middle cerebral artery. Ninety minutes after MCAo, the monofilament was carefully removed. The next day after MCAo surgery, acute neurological assessments were performed on the MCAo-induced rats to confirm neurological deficits. Before transplantation (7 days after MCAo induction), the rats with moderate to severe sensorimotor deficits (15 points or more in a modified neurological severity scale (mNSS) score) were selected for experiments. Of a total of 40 rats, 11 animals were not sued for several reasons, including death before transplantation (*n* = 4) and mild neurological deficits (*n* = 7). Therefore, a total of 29 rats were included in this study.

### 2.4. Intracerebral Transplantation of ep-iPSC-NPCs

One week after MCAo induction, rats were divided into three treatment groups: rats treated with ep-iPSC-NPC transplantation (ep-iPSC-NPC group, *n* = 9), rats treated with human fibroblast transplantation (fibroblast group, *n* = 10), and rats treated with only naïve culture medium (DMEM) as negative controls (vehicle group, *n* = 10). In the ep-iPSC-NPC group, a total of 2 × 10^5^ ep-iPSC-NPC was intracerebrally transplanted (Supplementary Fig. [Supplementary-material supplementary-material-1]). The cells were injected at two points (1 × 10^5^ cells at each point) that were coordinated as follows: (1) +1.6 mm anteroposterior (AP), -2.0 mm mediolateral (ML), and -3.0 mm dorsoventral (DV) and (2) +1.6 AP, -2.0 mm ML, and -6.0 mm dorsoventral DV from the bregma using a stereotaxic instrument with a speed of 0.5 *μ*L/min (Supplementary Fig. [Supplementary-material supplementary-material-1]). The needle was left for 5 min before retraction. In the fibroblast group, human foreskin fibroblasts (American Type Culture Collection, Manassas, VA, USA; CRL-2522), using the same number as for ep-iPSC-NPCs, were transplanted into the same coordinates. Cyclosporine A (CsA) was administered intraperitoneally to all animals (10 mg/kg on the previous day of transplantation and 5 mg/kg daily for up to 12 weeks).

### 2.5. Behavioral Tests

A total of five behavioral tests were conducted in all tested groups (ep-iPSC-NPC group, *n* = 9; fibroblast group, *n* = 10; and vehicle group, *n* = 10) for up to 12 weeks. For the rotarod test [11], each animal was trained daily for three consecutive days before MCAo induction. The rotarod speed was gradually increased from 4 to 40 r.p.m. for 2 min. Each rat was placed on a rotarod wheel, and the time of persistence on the cylinder was recorded. We recorded the time it took for each rat to fall down from the rotating wheel and calculated the average time from three trials. The tests were conducted 1 day before MCAo (pre) and once per week for up to 12 weeks after MCAo induction.

For the mNSS test, each rat was tested 1 day before MCAo induction and weekly for up to 12 weeks after MCAo induction. Each rat was given a score, which was the sum of each motor and sensory score. The highest mNSS score (28 points) represented the most severe motorsensory dysfunction whereas the minimum score (0 points) represented normal sensorimotor function.

For stepping tests [26], each rat was located vertically above a 5~7 cm treadmill. The speed of the treadmill was kept at 23 r.p.m. with a forward and backward direction. We counted the number of rats placing their forepaw on the belt. Each paw and direction were measured in three trials. All animals were examined weekly for 12 weeks.

For the staircase test [27], rats were placed on a diet during training and were trained 3 days before MCAo surgery using a staircase cage. In the cage, rats were trained to pick up pellets from stairs on the left. Every training step was performed in the dark, and rats were selected that ate more than 15 sugar pellets. After MCAo surgery, the test was performed biweekly, three times per test, for 12 weeks. The number of pellets each rat ate was counted.

For the apomorphine-induced rotation test [28], apomorphine (Sigma, 1.0 mg/kg in 0.2% ascorbate) was injected intraperitoneally into each rat. Each rat was placed in an acrylic box cage for 5 min, and the number of rotations was counted for 1 h. Rotations were defined as completing a circle of 360° toward the ipsilateral side of an injury. This test was performed biweekly for 12 weeks. This study was expressed as a percentage based on 0-week data.

### 2.6. Immunocytochemistry

To analyze the marker expression of undifferentiated human ep-iPSCs and ep-iPSC-NPCs, 12 mm^2^ glass coverslips were coated using iMatrix-511 for undifferentiated ep-iPSCs and PLO/LN for NPCs. Cells were seeded on coated 12 mm^2^ coverslips and cultured for 3 to 4 days. Cells on coverslips were washed three times with phosphate-buffered saline (PBS) containing calcium and magnesium (PBS^+^) and fixed in 4% paraformaldehyde for 15 min at room temperature. Fixed cells were washed three times with PBS^+^ and three times with 0.1% Triton X-100 in PBS. In the case of surface markers, fixed cells were washed in PBS without Triton X-100. Cells were then incubated in 5% normal horse serum (NHS) for 30 min. Afterwards, the samples were incubated with primary antibodies diluted in PBS overnight at 4°C: anti-OCT4 (1 : 500; Santa Cruz Biotechnology, Dallas, TX, USA; SC-5279), anti-SOX2 (1 : 100; Millipore, Burlington, MA, USA; AB5603), anti-SSEA4 (1 : 100; Developmental Studies Hybridoma Bank, Iowa City, IA, USA; MC-813-70), anti-TRA-1-81 (1 : 250; Chemicon International, Temecula, CA, USA; MAB4381), anti-TRA-1-60 (1 : 100; BD Biosciences, Franklin Lakes, NJ, USA; 560071), and anti-Nestin (1 : 200; R&D Systems, Minneapolis, MI, USA; AB1259).

The next day, the cells were washed with PBS and then incubated in the dark with secondary antibodies diluted in PBS for 2 h as follows: Alexa Fluor 555 goat-mouse IgG (1 : 250; Molecular Probes, Eugene, OR, USA; A21427), Alexa Fluor 555 goat anti-mouse IgM (1 : 250; Molecular Probes; A21726), Alexa Fluor 488 goat-rabbit IgG (1 : 250; Invitrogen, Carlsbad, CA, USA), and Alexa Flour 555 donkey anti-goat IgG (1 : 250; Life Technologies, Carlsbad, CA, USA; A21432). After 2 h, the cells were stained with 4′,6-diamidino-2-phenylindole (DAPI; 1 : 1000; Roche Diagnostics, Mannheim, Germany). Fluorescent images were captured using a confocal laser-scanning microscope imaging system (LSM510; Carl Zeiss Microimaging, Inc., Jena, Germany) and an ApoTome Microscope (Carl Zeiss Microimaging, Inc.).

### 2.7. Polymerase Chain Reaction

For reverse transcription polymerase chain reaction (RT-PCR) analysis of human ep-iPSCs and ep-iPSC-NPCs, total RNAs were isolated from cell pellets using Trizol (Invitrogen, Carlsbad, CA, USA; 15596-026). Complementary DNAs (cDNAs) were then synthesized from extracted total RNA using a cDNA synthesis kit (Cosmo Genetech, Seoul, Korea; CMRTK002). Pre-PCR was conducted at 42°C for 1 h and at 70°C for 10 min. RT-PCR was performed using i-Taq DNA polymerase (iNtRON Biotechnology, Seongnam, Korea; 25022) in a final volume of 20 *μ*L containing 100 ng/*μ*L cDNA for each sample. The following primers were used: GAPDH (forward primer: TGA CCA CAG TCC ATG CCA TCA CTG C; reverse primer: GTC ATA CCA GGA AAT GAG CTT GAC A); Oct3/4 (forward primer: CTG AAG CAG AAG AGG ATC AC; reverse primer: GAC CAC ATC CTT CTC GAG CC); NANOG (forward primer: TTC TTG ACT GGG ACC TTG TC; reverse primer: GCT TGC CTT GCT TTG AAG CA); SOX2 (forward primer: GCT GCA AAA GAG AAC ACC AA; reverse primer: CTT CCT GCA AAG CTC CTA CC); Lin28 (forward primer: CAC CAT GGG CTC CGT GTC CAA CCA GCA G; reverse primer: TCA ATT CTG TGC CTC CGG GAG CAG GGT AGG); Nestin (forward primer: TCC AGA AAC TCA AGC ACC A; reverse primer: AAA TTC TCC AGG TTC CAT GC); and Musashi (forward primer: ACA GCC CAA GAT GGT GAC TC; reverse primer: CCA CGA TGT CCT CAC TCT CA). First, denaturation was performed at 94°C for 5 min. Then, the annealing step was adjusted according to each marker, and the final extension step was performed at 72°C for 7 min. PCR cycling was carried out for 30 cycles. PCR products were detected using a 1.5% agarose gel containing ethidium bromide and visualized with a Gel Doc EZ system (Bio-Rad, Hercules, CA, USA).

### 2.8. Fluorescence-Activated Cell Sorting

When ep-iPSCs reached approximately 80% confluency, we conducted flow cytometry. The cultures were dissociated into a single-cell suspension using 0.5x TrypLE solution consisting of TrypLE Select Enzymes (Thermo Fisher Scientific) and 0.5 mM UltraPure EDTA (Thermo Fisher Scientific). Cells remained unfixed and were stained with phycoerythrin-conjugated antigen-specific antibodies of corresponding isotypes using the manufacturer's recommended concentrations. We used anti-SSEA4 (1 : 200) and anti-IgM isotype (1 : 200) and anti-TRA-1-60 (1 : 200) and anti-IgG3 isotype (1 : 200; all from BD Biosciences). After staining, the required amounts of samples were processed through a BD FACS Calibur™ flow cytometer (BD Biosciences). Data were obtained and analyzed using BD fluorescence-activated cell sorter (FACS) Diva software.

### 2.9. Immunohistochemistry

Thirteen weeks after MCAo surgery (i.e., 12 weeks after transplantation), rats were sacrificed and transcardially perfused with heparinized saline (0.9% NaCl), followed by 4% paraformaldehyde. After brains were extracted, the samples were fixed by 4% paraformaldehyde overnight at 4°C. The next day, the specimens were dipped into a 30% sucrose solution on a shaking device for 1 to 3 days at 4°C. The specimens were frozen in optimal cutting temperature compound (Leica Biosystems, Nussloch, Germany; 3801480). Double-label immunofluorescence staining was performed on free-floating 40 *μ*m sections. After staining, sections were washed three times in PBS for 5 min. Then, sections were incubated three times in PBS containing 0.3% Triton X-100 (Sigma) for 10 min. The sections were then incubated in 5% NHS (Vector Laboratories, Burlingame, CA, USA) in PBS containing 0.3% Triton X-100 for 1 h at room temperature. The following primary antibodies were incubated in 2% NHS in PBS containing 0.3% Triton X-100 (Sigma) overnight at 4°C: anti-human specific nuclei (hNu), anti-human mitochondria (hMito, MTC02), anti-human specific Nestin (hNestin), anti-microtubule-associated protein-2 (MAP2), anti-glutamate decarboxylase 65&67 (GAD65/67), anti-medium-sized-spiny neuronal marker (Darpp32), anti-gamma aminobutyric acid (GABA), anti-synaptophysin- (SVP-) 38, anti-O4, anti-glial fibrillary acidic protein (GFAP), anti-brain-derived neurotrophic factor (BDNF), anti-doublecortin (DCX), anti-proliferating nuclear antigen (PCNA), anti-polysialic acid-neural cell adhesion molecule (PSA-NCAM), anti-ED1, anti-Iba-1, anti-inducible nitric oxide synthase (iNOS), anti-CD206, and anti-caspase 3. Detailed information on individual primary antibodies used in immunohistochemistry is described elsewhere (Supplementary [Supplementary-material supplementary-material-1]). The next day, the sections were washed with PBS and incubated with fluorescent dye-conjugated secondary antibodies against the relevant species for 2 h at room temperature. Afterwards, sections were counterstained with DAPI. Fluorescent-labeled specimens were visualized under a confocal laser-scanning microscope (LSM510; Carl Zeiss Microimaging Inc., München, Germany).

For 5′-bromo-2′-deoxyuridine (BrdU) staining, BrdU (50 mg/kg, Sigma-Aldrich) was injected intraperitoneally 1 week before euthanasia. BrdU-positive cells were detected by immunohistochemistry using an antibody against BrdU after denaturation of DNA by treatment with 1 M HCl for 30 min at 37°C. Terminal deoxynucleotidyl transferase-mediated dUTP nick-end labeling (TUNEL) assays were performed using an *In Situ* Cell Death Detection Kit (Roche, Indianapolis, IN, USA) according to the manufacturer's instructions.

One month before euthanasia, 1 *μ*L of fluorogold (FG, 4% solution in sterile saline; Molecular Probes) was injected into the globus pallidus (-1.3 mm AP, -3.4 mm ML, and -6.5 mm DV from the bregma) ipsilateral to the cell transplantation. Stereotaxic injection was made using a 26 G Hamilton syringe-injection pump for 5 min. One week later, FG-injected animals were sacrificed and brain tissues extracted.

### 2.10. Western Blot

We evaluated the expression of BDNF of ep-iPSC-NPC compared to human fibroblasts in normoxic or hypoxic conditions. In hypoxic experiment, we induced mild hypoxia (3% O_2_) to cells as the time of ep-iPSC-NPC transplantation in this study corresponds to a mild hypoxic period (7 days after ischemia-reperfusion). The 1 × 10^5^ ep-iPSC-NPCs and human fibroblasts were incubated under normoxic condition consisting of humidified 95% air/5% CO_2_ for 24 h. After then, the cells were exposed to 3% O_2_ for 24 h in a hypoxic chamber (Galaxy 48R CO_2_ incubator, Eppendorf, Hamburg, Germany). After hypoxia, the cells were returned to normoxic conditions for 24 h. The controls were continuously maintained under normoxic condition for the full duration.

The cells were lysed using PRO-PREP™ (iNtRON Biotechnology, Seongnam, Korea) for 20 min on ice. The protein concentrations were confirmed using Pierce™ BCA Protein Assay kit (Thermo Scientific, Waltham, MA, USA), then were boiled for 5 min at 95°C. The protein was separated using 12% (*w*/*v*) sodium dodecyl sulfate-polyacrylamide gel electrophoresis gel and transferred to Immobilon-P polyvinylidene fluoride membrane (Millipore, Carrigtwohill, County Cork, Ireland). The membranes were incubated with primary antibodies BDNF (1 : 1000, Abcam, Cambridge, MA, USA) and *β*-actin (1 : 1000, Santa Cruz Biotechnology, Santa Cruz, CA, USA) in 2% skim milk in TBST overnight at 4°C. All bands were developed using chemiluminescence reagent (Clarity™ ECL substrate, Bio-Rad, Hercules, California) and were detected on G:BOX Chemi XX6 gel doc system (Syngene, Frederick, USA). Analysis was performed using ImageJ software (ImageJ; National Institutes of Health, Bethesda, MD, USA).

### 2.11. Infarct Size Measurement

Cresyl violet staining on 12 *μ*m coronal sections was performed to measure the final infarct size. A total of eight serial sections were analyzed in each animal. We estimated the infarct size as a percentage of the intact contralateral hemisphere by the use of the following equation: estimated infarct size (%) = [1–(area of remaining ipsilateral hemisphere/area of intact contralateral hemisphere)] × 100. The areas of interest were measured with the use of ImageJ software, and the values were summed for six serial coronal sections per brain.

### 2.12. Motor-Evoked Potential Analysis

Motor-evoked potential (MEP) analysis was performed 12 weeks after cell transplantation. All animals were anesthetized and fixed in a stereotaxic apparatus. Craniotomy in the shape of a square (bregma based on the right to 5 mm, caudal 5 mm) was undertaken using a drill, and the electrode was placed in the right frontoparietal area through the square. To induce MEP, the animals were stimulated by a 0.2 ms pulse duration and shocked with a current of 6 mA intensity by a stimulus isolator (A365; World Precision Instruments, Sarasota, FL, USA) and pulse generator (A365, World Precision Instruments). The recording needle electrode was placed in the forelimb and hindlimb muscles. The amplitude of each group was compared by SonuView program.

### 2.13. Grafted Cell Migration and Differentiation

The engrafted cells were measured by counting hNu immunostains using 3,3′-diaminobenzidine (DAB) staining (SK-4100; Vector Laboratories, Burlingame, CA, USA) in five coronal brain sections each of a 40 *μ*m thickness. Cell counts were done by semiautomated particle analysis using ImageJ software (NIH) by investigators blinded to behavioral results. Because of a high variability in the engraftment rate across animals and the difficulty in cell counts of differentiated cells in whole brain sections, the average proportion of differentiated cells could not be evaluated. Instead, we descriptively analyzed the maximum proportion of each differentiated cell.

### 2.14. Statistical Analysis

Statistical analyses were conducted using a Statistical Analysis System program (Enterprise 4.1; SAS, Seoul, Korea). All values are presented as a mean ± standard error of the mean (SEM). Tissue analysis measurements were analyzed using a one-way analysis of variance (ANOVA), and behavior test performances were analyzed by two-way ANOVA. Statistical significance was considered at *p* < 0.05.

## 3. Results

### 3.1. Efficient Differentiation of ep-iPSCs into NPCs

**Figure 1:**
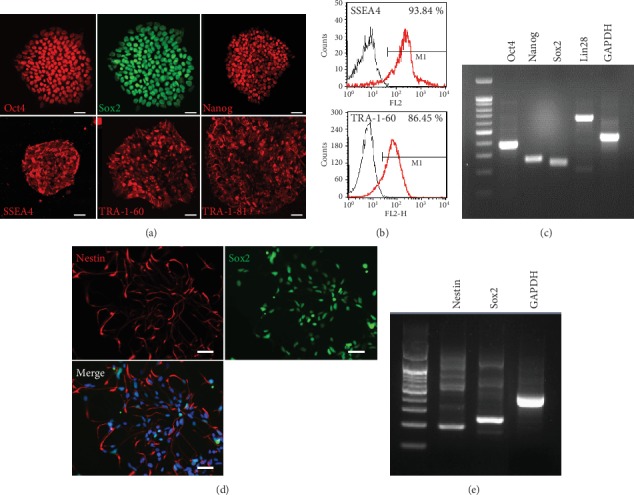
Neural induction of human ep-iPSCs into neural precursor cells. (a) Immunofluorescence staining of undifferentiated markers (OCT4, SOX2, NANOG, SSEA4, TRA-1-60, and TRA-1-81) in induced pluripotent stem cells (ep-iPSCs). (b) Fluorescent-activated cell sorting (FACS) analysis of undifferentiated markers (SSEA4 and TRA-1-60) in ep-iPSCs. (c) Polymerase chain reaction (PCR) of undifferentiated markers (OCT4, NANOG, SOX2, and LIN28) in ep-iPSCs. (d) Double immunofluorescence staining for neural precursor cell (NPC) markers (Nestin and SOX2) in ep-iPSC-NPCs. DAPI (blue) stain nuclei. (e) PCR analysis of NPC markers (Nestin and SOX2) in ep-iPSC-NPCs. Scale bars: 50 *μ*m. ep-iPSC-NPCs: neural precursor cells differentiated from induced pluripotent stem cells.

First, we characterized ep-iPSCs by immunocytochemistry, PCR, and FACS analysis. In immunocytochemical analysis, ep-iPSCs showed strong positive staining for pluripotency markers, such as OCT4, SOX2, and NANOG (Figure 1(a)). The cells also showed strong positive staining for SSEA4, TRA-1-60, and TRA-1-81. FACS analysis revealed that nearly 90% of cells showed positive staining for SSEA4 and TRA-1-60 (Figure 1(b)). PCR analysis showed strong gene expression of *OCT4*, *NANOG*, *SOX2*, and *LIN28* (Figure 1(c)). In karyotypic analysis, ep-iPSCs did not show any chromosomal abnormalities (data not shown).

We next performed neural induction to generate ep-iPSC-NPCs by the use of dual SMAD inhibition under feeder-free and xeno-free conditions. After the completion of the NPC induction of ep-iPSCs, immunohistochemistry and PCR analysis were conducted to characterize ep-iPSC-NPCs. The cells exhibited strong expression of Nestin and SOX2 in immunohistochemistry (Figure 1(d)) and PCR (Figure 1(e)), suggesting that NPCs were successfully differentiated from ep-iPSCs.

### 3.2. Functional Recovery of MCAo Rats after ep-iPSC-NPC Transplantation

**Figure 2:**
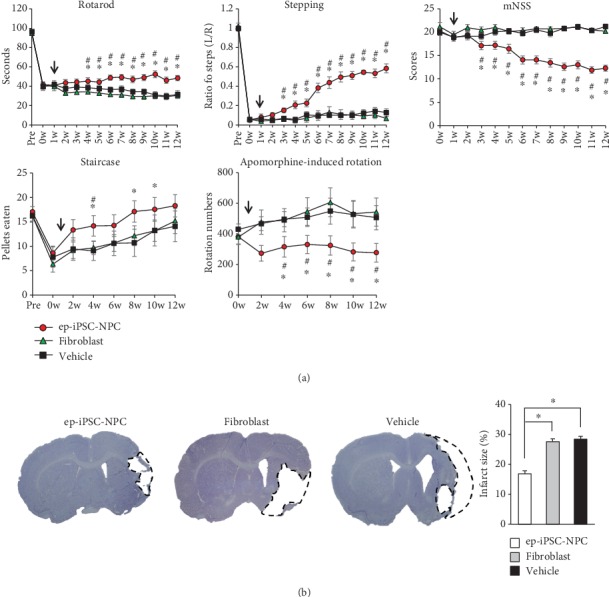
Behavioral tests and infarct size measurements in MCAo rats after ep-iPSC-NPC transplantation. (a) Behavioral tests of the ep-iPSC-NPC (*n* = 9), fibroblast (*n* = 10), and vehicle groups (*n* = 10) over 12 weeks. Statistical analyses were conducted by two-way ANOVA. ^∗^*p* < 0.05 between the ep-iPSC-NPC and fibroblast groups, ^#^*p* < 0.05 between the ep-iPSC-NPC and vehicle groups. Arrow indicates transplantation. (b) Measurement of infarct size in the ep-iPSC-NPC (*n* = 5), fibroblast (*n* = 5), and vehicle groups (*n* = 5). The infarct area (black dashed line) was measured on eight serial Cresyl violet-stained coronal sections. Data are shown as mean ± standard error of the mean (SEM), ^∗^*p* < 0.05 by one way ANOVA. ep-iPSC-NPCs: neural precursor cells differentiated from induced pluripotent stem cells; MCAo: middle cerebral artery occlusion.

To evaluate whether the transplantation of ep-iPSC-NPCs could induce functional effects in stroke, the behaviors of rats in the ep-iPSC-NPC group were compared to those in the fibroblast and vehicle groups. In the rotarod test, rats in the ep-iPSC-NPC group endured for a longer time period on the rotarod cylinder than rats in the fibroblast and vehicle groups from 3 to 11 weeks after ep-iPSC-NPC transplantation (Figure 2(a)). Rats in the ep-iPSC-NPC group, compared to those in the fibroblast and vehicle groups, exhibited a significant improvement in the stepping test and mNSS scores from 2 weeks to 11 weeks after ep-iPSC-NPC transplantation (Figure 2(a)). The staircase test and apomorphine-induced rotation test were performed biweekly. However, on the staircase test, the ep-iPSC-NPC group showed a significant improvement only 3, 7, and 9 weeks after transplantation compared to other groups (Figure 2(a)). In the apomorphine-induced rotation test, rats of the ep-iPSC-NPC group had a lower rotation number ratio from 3 to 11 weeks after transplantation compared those of the other groups (Figure 2(a)). In addition to functional recovery, the final infarct size 11 weeks after transplantation was significantly decreased in rats of the ep-iPSC-NPC group compared to those of the fibroblast and vehicle groups (Figure 2(b)). These findings indicate that ep-iPSC-NPC transplantation induces a gradual improvement in neurological deficits in stroke-damaged rats.

### 3.3. Differentiation Capacity of Grafted ep-iPSC-NPCs into Neuronal and Glial Lineages in MCAo Rats

To identify the engraftment of ep-iPSCs-NPCs in the brain, we performed immunohistochemistry for hNu. Twelve weeks after transplantation, most transplanted cells were detected in the injection area while a small proportion of transplanted cells was sparsely present in the peri-infarct area (Supplementary Fig. [Supplementary-material supplementary-material-1]). The rate of engraftment was highly variable across the tested animals. The average number of hNu-positive cells was 4565 ± 4969, that is, approximately 2.3% of the initial number of transplanted cells. The maximal engraftment rate was 7.5% (approximately 15,000 hNu-positive cells). The grafted cells did not form tumor-like structures at the injection sites or around the peri-infarct lesion in all examined animals 12 weeks after transplantation.

**Figure 3:**
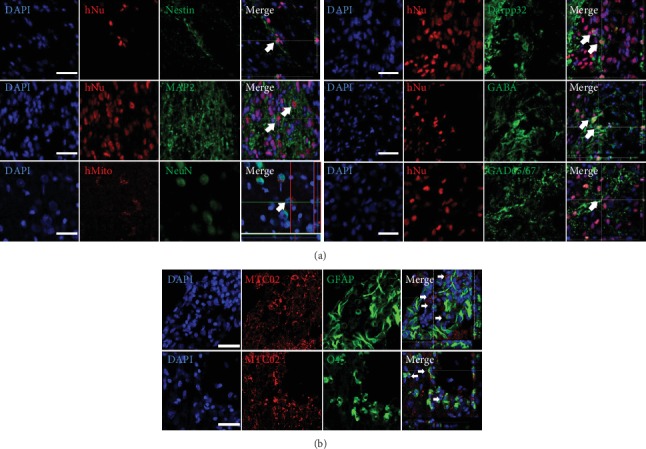
Differentiation of grafted ep-iPSC-NPCs in the brains of MCAo rats. (a) Double immunofluorescence staining for neuronal differentiation markers (MAP2, NeuN, Darpp32, GABA, and GAD65/67) in ep-iPSC-NPC-transplanted brain. Human-specific nuclear (hNu) and mitochondrial (hMito) markers were used to detect grafted cells. (b) Double immunofluorescence staining for glial differentiation markers (GFAP and O4) in the ep-iPSC-NPC-transplanted brain. The human mitochondrial marker (MTC02) was used to detect grafted cells. The human-specific marker (hNu) was used to detect grafted cells. All samples were counterstained with DAPI. Scale bars: 50 *μ*m. ep-iPSC-NPCs: neural precursor cells differentiated from induced pluripotent stem cells; MCAo: middle cerebral artery occlusion.

To identify whether grafted ep-iPSC-NPCs became differentiated into neuronal or glial lineages, we performed double immunohistochemical staining for neuronal and glial markers. Both human-specific markers (hNu, hMito, and MTC02) and neuronal or glial markers were costained to examine whether cells were of human origin. The majority of transplanted cells strongly expressed Nestin at the injection site 12 weeks after transplantation (Supplementary Fig. [Supplementary-material supplementary-material-1]). Less than 10% of hNu-positive cells showed staining with antibodies against mature neuronal markers (MAP2 and NeuN) at the graft site or adjacent to the infarct boundary zone (Figure 3(a)). A small proportion of grafted cells (less than 3% of hNu-positive cells) showed staining with antibodies against Darpp32, GABA, and GAD65/67 (Figure 3(a)). The grafted cells were also stained with antibodies against astrocyte (GFAP) and oligodendrocyte (O4) markers, although the numbers of stained cells were extremely low (Figure 3(b)).

In addition, grafted cells strongly expressed BDNF, a potent neurotrophic factor that induces neuronal differentiation, in the peri-infarct boundary zone (Supplementary Fig. [Supplementary-material supplementary-material-1]). ep-iPSC-NPCs showed higher expression of BDNF protein under both normoxic and mild hypoxic conditions compared to fibroblasts (Supplementary Fig. [Supplementary-material supplementary-material-1]). There was no difference in BDNF expression between ep-iPSC-NPCs under normoxic condition and those under hypoxic condition.

### 3.4. Functional Connectivity of Grafted ep-iPSC-NPCs with the Host Brain in MCAo Rats

**Figure 4:**
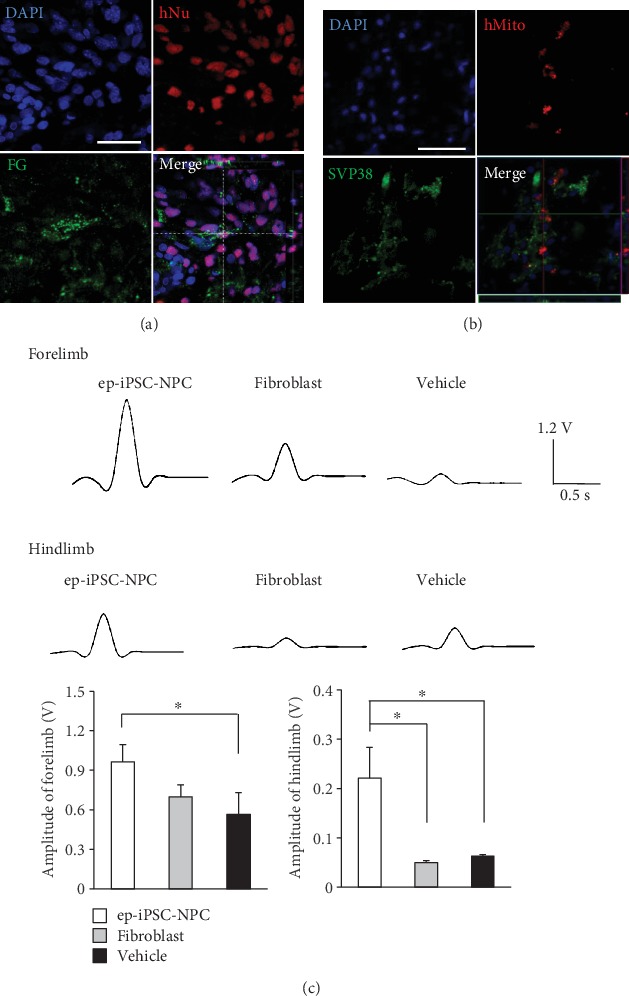
Functional connectivity of grafted ep-iPSC-NPCs with host brain in MCAo rats. (a) Double immunofluorescence staining of fluorogold (FG) in the ep-iPSC-NPC-transplanted brain. A human-specific marker (human-specific nuclei (hNu)) was used to detect grafted cells. Merged cells suggested that grafted cells connected with host tissue. (b) Double immunofluorescence staining of SVP-38, a synaptic vesicle marker, in the ep-iPSC-NPC-transplanted brain. A human-specific marker (human mitochondria (hMito)) was used to detect grafted cells. All samples were counterstained with DAPI. Scale bars: 50 *μ*m. (c) Motor-evoked potentials (MEP) of paretic limbs in rats of the ep-iPSC-NPC, fibroblast, and vehicle groups (*n* = 3 in each group). Data are shown as mean ± standard error of the mean (SEM), ^∗^*p* < 0.05 by one-way ANOVA. ep-iPSC-NPCs: neural precursor cells differentiated from induced pluripotent stem cells; MCAo: middle cerebral artery occlusion; SVP: synaptophysin.

To examine the functional connectivity of grafted ep-iPSC-NPCs with the host brain, we first performed a retrograde neuronal tracer analysis using FG (Figure 4(a)). After behavior testing for 12 weeks, three animals in the ep-iPSC-NPC group were injected with FG in the globus pallidus. FG- and hNu-double positive staining cells were observed in the peri-infarct boundary zone. This suggests that grafted ep-iPSC-NPCs connect with the host tissue and may contribute to the formation of host neural networks. In addition, a proportion of the grafted cells showed copositive staining for SVP-38, a presynaptic vesicle marker (Figure 4(b)), indicating that grafted cells participated in synaptic transmission.

To evaluate neuronal functions in ep-iPSC-NPC transplantation, a MEP study was performed in all groups (*n* = 3 per group) (Figure 4(c)). In the vehicle group, the MEP amplitude was hardly detected in the paretic limbs of rats. Differences in MEP amplitudes between animals of the vehicle and fibroblast groups were not found. In contrast, the MEP amplitude in the forelimb of rats in the ep-iPSC-NPC group was higher than that of rats in the vehicle group. The MEP amplitude in the hindlimb of rats in the ep-iPSC-NPC group was higher than that of rats in the vehicle and fibroblast groups. The enhanced MEP in the ep-iPSC-NPC group indicates an improvement in neuronal networks in the damaged rat brain after ep-iPSC-NPC transplantation.

### 3.5. Enhanced SVZ Neurogenesis after ep-iPSC-NPC Transplantation in MCAo Rats

**Figure 5:**
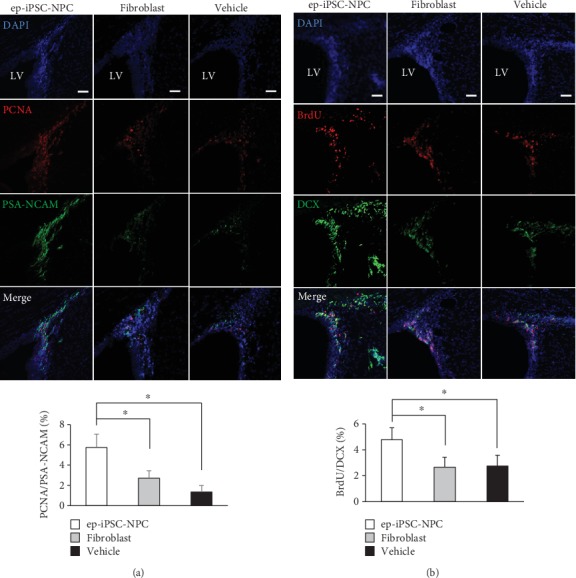
Subventricular zone neurogenesis analyses in MCAo rats with ep-iPSC-NPC transplantation. (a) Double immunofluorescence staining of PCNA/PSA-NCAM in the SVZ of rats in the ep-iPSC-NPC, fibroblast, and vehicle groups (*n* = 5 in each group). (b) Double immunofluorescence staining of BrdU/DCX in the SVZ of the ep-iPSC-NPC, fibroblast, and vehicle groups (*n* = 5 in each group). LV indicates lateral ventricle. All samples were counterstained with DAPI (blue). Scale bars: 50 *μ*m. Data are shown as mean ± standard error of the mean (SEM), ^∗^*p* < 0.05 by one-way ANOVA. BrdU: 5′-bromo-2′-deoxyuridine; DCX: doublecortin; ep-iPSC-NPCs: neural precursor cells differentiated from induced pluripotent stem cells; MCAo: middle cerebral artery occlusion; PCNA: proliferating nuclear antigen; PSA-NCAM: polysialic acid-neural cell adhesion molecule; SVZ: subventricular zone.

We next investigated the endogenous effect of grafted ep-iPSC-NPCs in MCAo rats. First, we investigated changes in neurogenesis in the subventricular zone (SVZ) in the damaged brain after ep-iPSC-NPC transplantation. To evaluate SVZ neurogenesis, double immunofluorescence staining for markers of proliferating and migrating neural precursors (PCNA/PSA-NCAM and BrdU/DCX) was performed in the SVZ of rats in the three tested groups (*n* = 5 per group). In PCNA/PSA-NCAM double immunofluorescence staining, the numbers of PCNA/PSA-NCAM copositive cells were significantly higher in tissues from rats in the ep-iPSC-NPC group than from other groups (Figure 5(a)). The numbers of BrdU/DCX copositive cells were significantly higher in the ep-iPSC-NPC group than the other groups (Figure 5(b)). These findings suggest that ep-iPSC-NPCs promote SVZ neurogenesis in the damaged brain following stroke via a paracrine mechanism.

### 3.6. Decrease of Poststroke Inflammation and the Promotion of Wound Healing after ep-iPSC-NPC Transplantation in MCAo Rats

**Figure 6:**
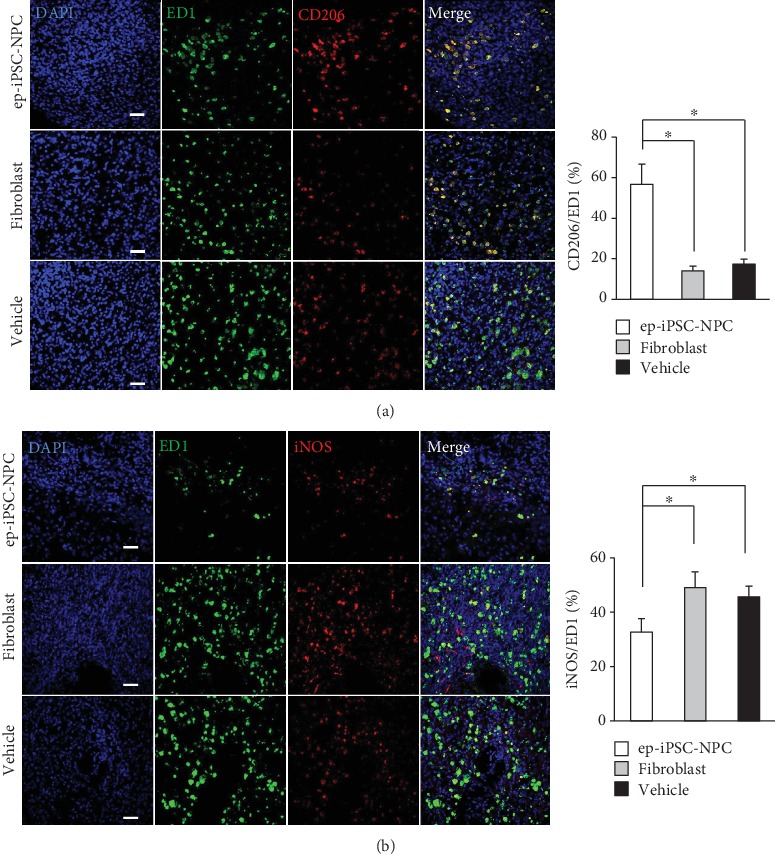
Poststroke inflammation analyses in MCAo rats with ep-iPSC-NPC transplantation. (a) Double immunofluorescence staining of ED1/CD206 in the brains of rats of the ep-iPSC-NPC, fibroblast, and vehicle groups (*n* = 5 in each group). (b) Double immunofluorescence staining of ED1/iNOS in the brains of rats of the ep-iPSC-NPC, fibroblast, and vehicle groups (*n* = 5 in each group). All samples were counterstained with DAPI (blue). Scale bars: 50 *μ*m. Data were shown as mean ± standard error of the mean (SEM), ^∗^*p* < 0.05 by one-way ANOVA. ep-iPSC-NPCs: neural precursor cells differentiated from induced pluripotent stem cells; iNOS: inducible nitric oxide synthase; MCAo: middle cerebral artery occlusion.

To investigate changes in the inflammatory response in the damaged brain after ep-iPSC-NPC transplantation, immunofluorescence staining for microglial markers (ED1 and Iba-1) was performed in the three tested groups (*n* = 5 per group). In the vehicle group, numerous ED1- and Iba-1-positive cells were found in the peri-infarct area. The numbers of ED1- and Iba-1-positive cells were not different in the fibroblast group compared to the vehicle group. In contrast, the numbers of ED1- and Iba-1-positive cells were significantly lower in the ep-iPSC-NSC group than in the other groups (Supplementary Fig. [Supplementary-material supplementary-material-1]). We next investigated the proportion of different microglial phenotypes using double immunofluorescence staining for M1-like (iNOS/ED1) and M2-like (CD206/ED1) microglia. The proportion of CD206/ED1 copositive cells was found to be higher (Figure 6(a)) whereas the proportion of iNOS/ED1 copositive cells was lower in the ep-iPSC-NPC group compared to the fibroblast and vehicle groups (Figure 6(b)). These findings suggest that ep-iPSC-NPCs not only decreased poststroke inflammation but also promoted the healing process in the damaged brain.

### 3.7. Decrease of Astroglial Scar Formation and Apoptosis after iPSC-NPC Transplantation in MCAo Rats

**Figure 7:**
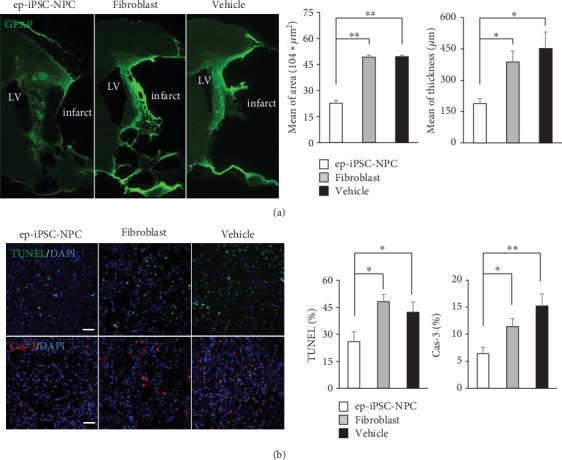
Glial scar formation and apoptosis analyses in MCAo rats with ep-iPSC-NPC transplantation. (a) Immunofluorescence staining of glial fibrillary acidic protein (GFAP) in the brains of rats in the ep-iPSC-NPC, fibroblast, and vehicle groups (*n* = 5 in each group). LV indicates the lateral ventricle. (b) Immunofluorescence staining of TUNEL (upper panel) and caspase-3 (lower panel) in the brains of rats of the ep-iPSC-NPC, fibroblast, and vehicle groups (*n* = 5 in each group). All samples were counterstained with DAPI (blue). Scale bars: 50 *μ*m. Data were shown as mean ± standard error of the mean (SEM), ^∗^*p* < 0.05 by one-way ANOVA. ep-iPSC-NPCs: neural precursor cells differentiated from induced pluripotent stem cells; MCAo: middle cerebral artery occlusion; TUNEL: terminal deoxynucleotidyl transferase-mediated dUTP nick-end labeling.

To investigate changes in astroglial scar formation, one of the hazardous factors for neuronal regeneration following stroke, in the damaged brain after ep-iPSC-NPC transplantation, immunofluorescence staining for astrogliosis markers (GFAP) was performed in the three tested groups (*n* = 5 per group) (Figure 7(a)). In the vehicle group, a large area of GFAP-positive glial scar was found in the area of the infarct. The mean area and thickness of the GFAP-positive area did not differ between the vehicle and fibroblast groups. Interestingly, both the mean area and thickness of the GFAP-positive area were significantly lower in the ep-iPSC-NPC group compared to the other groups. This finding suggests that ep-iPSC-NPC prevented astroglial scar formation following stroke.

To investigate changes in neuronal cell death in ep-iPSC-NPSC transplantation, immunofluorescence staining for TUNEL and caspase-3 at the peri-infarct area in each MCAo rat was performed in the three tested groups (*n* = 5 per group) (Figure 7(b)). The numbers of TUNEL- and caspase-3-positive cells were not different in the fibroblast group compared to the vehicle group. In contrast, the numbers of TUNEL- and caspase-3-positive cells were significantly lower in the ep-iPSC-NSC group compared to the other groups. These findings indicate that ep-iPSC-NPC transplantation prevents ongoing programmed cell death in the damaged brain following stroke.

## 4. Discussion

The present study demonstrated the therapeutic potential of ep-iPSC-NPCs in an animal stroke model. Animals transplanted with ep-iPSC-NPCs exhibited a consistent improvement in various sensorimotor and behavioral functional tests. Some of the grafted ep-iPSC-NPCs had the potential to differentiate into neuronal and glial lineages. In addition, the grafted cells enhanced endogenous brain repair such as SVZ neurogenesis and the healing of poststroke inflammation and glial scar formation.

We showed the therapeutic potential of iPSCs transducing reprogramming factors using EP delivery in an animal model of stroke. The ep-iPSCs used in the present study were made under clinically compatible conditions for future medical applications. They were generated using nonviral EP containing a combination of five reprogramming factors (OCT3/4, KLF4, SOX2, L-Myc, and Lin28) for the highly efficient induction of iPSCs [23]. Furthermore, the cells were obtained from peripheral blood, an easily accessible source compared to other tissues. The cells were maintained under xeno- and feeder-free conditions, which enabled us to use ep-iPSCs safely without concern of contamination by animal-origin products. Another common integration-free method is to use Sendai virus, which does not cause genomic integration. However, the use of Sendai virus is limited in clinical applications due to the presence of viral particle remnants and therefore the risk of infection. In comparison, an EP-based reprogramming method is a nonviral method for the generation of iPSCs that avoids the risk of viral contamination. In addition, the vectors contained a component of the Epstein–Barr virus, EBNA1, which is known to induce plasmid amplification, which enables the relatively high and long-term expression of reprogramming factors [22, 23], indicating a highly efficient method for iPSC generation. Therefore, we suggest that ep-iPSCs may be a leading candidate for future clinical studies [29].

In this study, transplantation of ep-iPSC-NPCs into the stroke-damaged brain led to a consistent improvement across sensorimotor and behavioral tests. Previous studies demonstrated that iPSC-NPC transplantation restored sensorimotor and behavioral function in stroke-damaged animals in various tests including the rotarod, staircase, corridor, beam walking, locomotor activity, adhesive removal, and Morris water maze tests [15, 19–21, 24]. Although most studies undertook behavioral tests during a short period (about 3~4 weeks) [19–21, 24], several studies showed a significant behavioral improvement over a long-term period [15, 18]. In the present study, ep-iPSC-NPC-transplanted animals showed a gradual improvement in their functional deficit during the 11 weeks after transplantation, especially in rotarod, mNSS, and stepping tests. Some studies have used iPSC-NPCs transplanted during the acute phase of stroke [19, 21], and other studies found a functional recovery in stroke animals transplanted with iPSCs-NPCs in the subacute phase of stroke (1 week after stroke induction) [15, 20, 24]. In the present study, ep-iPSC-NPC transplantation in the subacute phase of stroke restored functional deficits in stroke animals. Furthermore, the MEP test demonstrated an improvement in motor deficits of paretic limbs after ep-iPSC-NPC transplantation. These findings indicate that ep-iPSC-NPC transplantation can give rise to long-term functional recovery in subacute stroke. This finding is particularly important for clinical translation because there is no effective treatment for this time phase of stroke. Therefore, based on this result and other previously reported studies, the use of ep-iPSC-NPCs may be a promising therapeutic option for patients with chronic stroke. One issue to address in this study is the natural course of behavior in stroke-damaged rats. Such animals usually show a spontaneous recovery following stroke. Although our stroke-damaged rats showed a partial improvement in the staircase test, the other four tests did not show a recovery. Although the exact reason for a lack of spontaneous recovery is not clear, one possible explanation is the inclusion of animals with severe functional deficits that usually have a poor functional outcome in an *in vivo* or clinical setting. Another issue to address is the effect of CsA on functional recovery. CsA administration induces NPC activation and neural induction [30, 31], although a contrasting result has also been reported [32]. The use of CsA is inevitable to prevent the immune rejection of xenografts. Although we cannot completely rule out the effect of CsA on transplanted NPCs in this study, we suggest that the functional improvement is mainly mediated by the transplanted cells rather than CsA as the vehicle, particularly since the HDF group that received CsA did not show any functional improvement.

One of the therapeutic mechanisms of ep-iPSC-NPC transplantation is neuronal replacement, which may be a prerequisite for functional recovery in stroke. NPCs are an attractive cell source for use in stroke therapy compared to other cell sources, such as MSCs that have shown no evidence for neuronal differentiation *in vivo*. In this study, a proportion of transplanted ep-iPSC-NPCs showed the potential to engraft and differentiate into various neuronal cell lineages, such as GABAergic neurons and medium-sized spiny neurons. Furthermore, grafted cells were functionally active and connected to the host brain, expressed a presynaptic vesicle marker and showed a striatal connection with host neurons. However, it should be noted that the survival and engraftment of transplanted cells was very low (2.3% of intimal transplanted cells). Previous studies have shown that the engraftment and differential potential of transplanted ESC- or iPSC-derived NPCs were highly variable. Approximately 5~30% of cells survive 4 weeks after transplantation, with the numbers of engrafted cells gradually decreasing thereafter [15, 17–20, 33–36]. Even if the cells had survived, the number of differentiated neurons among the grafted cells was low in this study (less than 10% of engrafted cells). Although the differential potential of transplanted NPCs varies across studies, about 5~70% of these cells differentiated into mature neurons but rarely differentiated into a glial lineage [15, 17–20, 33–36]. The variation in engraftment and the differential capacity of NPCs across studies can be accounted for by the use of differences in cell sources, amounts of transplanted cells, delivery route, and the timings of transplantation and histological examination. These different transplantation conditions may have influenced the interactions of transplanted cells with the microenvironment of the host brain, thus producing different results. In addition to engraftment, a very small proportion of surviving NPCs differentiated into mature neurons. Even rats that did not display a successful engraftment (no grafted cells detected) showed a similar functional recovery to those with a successful engraftment of transplanted cells. This suggests that cell engraftment is not necessary to maintain a functional recovery and thus another therapeutic mechanism(s) may exist [15, 35].

In the present study, the majority of transplanted cells were Nestin-positive, indicating that they still remained undifferentiated in the ischemic brain in the long term. This finding is consistent with previous studies showing that a significant proportion of engrafted NPCs remained as undifferentiated Nestin-positive NPCs in the ischemic brain [35, 36]. This phenomenon may be explained by their ability to retain their original characteristics or that the microenvironment may have arrested the capacity of the transplanted NPCs to differentiate into mature neurons or glia [37, 38]. Recent studies have suggested that nonproliferating, undifferentiated transplanted NPCs play a role in functional recovery because of a bystander effect on undifferentiated NPCs that release immunomodulatory and neurotrophic molecules [39–43].

Many researchers have shown that NPC transplantation enhances endogenous repair mechanisms through a paracrine mechanism, such as neovascularization [15] and SVZ neurogenesis [33, 44, 45]. Of these, SVZ neurogenesis is an endogenous process of neural repair under physiological conditions and is enhanced in pathological conditions, such as in ischemia. Endogenous NSCs in the SVZ proliferate, migrate into the injured area, and differentiate into functional neurons around the infarcted area for several months after a stroke [46]. Several studies have reported the promoting effect of NPC transplantation on SVZ neurogenesis [33, 44, 45]. In a study of human ESC-derived NPC transplantation in a distal MCAo model, DCX-positive cells increased in the SVZ by 74 days after post-MCAo surgery [44]. Transplantation of immortalized human NSCs in the ischemic rat brain increased the numbers of BrdU-positive cells and BrdU/DCX copositive cells in the SVZ [47]. We performed comprehensive analyses on SVZ neurogenesis using double-immunostaining (BrdU/DCX and PCNA/PSA-NCAM) and demonstrated that ep-iPSC-NPC-transplanted animals showed higher BrdU/DCX copositive cells and PCNA/PSA-NCAM copositive cells in the SVZ. Among the various trophic factors released by human NPCs, BDNF is highly enriched in such cells. BDNF promotes neuronal differentiation, neuronal survival, and dendrite outgrowth [48]. We found that grafted ep-iPSC-NPCs showed strong BDNF expression *in vitro* and in the ischemic brain, suggesting that BDNF is one of the ep-iPSC-NPC secreted factors that mediate functional recovery in ischemic stroke. The local intracerebral grafting of adult mouse NPCs in the brain parenchyma is associated with elevated brain concentrations of BDNF, fibroblast growth factor, and vascular endothelial growth factor in the subacute stroke phase in mice [49]. Such elevated growth factor levels were still present as late as 2 months after the intracerebral transplantation of NPCs transduced with heat shock protein [49]. A previous study showed that treatment with the conditioned medium of NPCs promoted SVZ neurogenesis following stroke [50], suggesting that SVZ neurogenesis can be mediated by NPCs secreting BDNF.

Another proposed mechanism of functional recovery by ep-iPSC-NPC transplantation is attenuating poststroke inflammation and subsequent glial scar formation. Following stroke, activated microglia and blood-borne macrophages secrete proinflammatory cytokines and subsequently stimulate astrocytes. Such poststroke inflammation and subsequent astrogliosis promote neuronal cell death and prevent neuronal regeneration during the healing stage after brain injury [51]. Several studies have demonstrated that transplantation of NPCs decreases the numbers of Iba-1- and/or ED1-positive microglia [11, 20, 33] and the expression of proinflammatory cytokines [43, 52]. This immunomodulatory action of NPCs is still observed when NPCs are systematically delivered in stroke animal models [43, 53], suggesting that the mechanism of its effect is mediated by the paracrine action of the cells. In the present study, we found that ED1-positive inflammatory cells were decreased in animals transplanted with ep-iPSC-NPCs. Furthermore, we observed that ep-iPSC-NPC transplantation changed the proportion of immunophenotypes among inflammatory cells. Transplantation of ep-iPSC-NPCs increased the proportion of anti-inflammatory M2-like cells (CD206-positive cells) whereas it decreased the proportion of proinflammatory M1-like cells (iNOS-positive cells) compared to controls. M2-like cells serve as repair machinery after stroke by stopping inflammation and promoting wound healing in stroke-induced damaged areas [54, 55]. A recent study showed that NPCs grafted into the injured spinal cord modulated the inflammatory microenvironment by reducing the number of M1-like cells [56]. While surviving long term next to blood vessels, undifferentiated NPCs establish cell-to-cell contacts with endogenous phagocytes via cellular-junctional coupling and alter the phenotypes of inflammatory cells (from an M1 to an M2 phenotype) [56]. Based on previous reports and our findings, ep-iPSC-NPC transplantation not only reduces the magnitude of the inflammatory process but also enhances the healing process through anti-inflammatory cells in the brain.

Reactive astrocytes and glial scarring are one of the most prominent pathological features in cerebral ischemia. These are an adaptive defense response that is both beneficial and detrimental to the injured central nervous system. In particular, early after ischemic injury, the main function of reactive astrocytes is to preserve the integrity of nervous tissue [57]. However, with time, the process becomes increasingly unregulated and maladaptive and progresses to form a compact glial scar. The glial scar further accentuates inflammation and generates a highly toxic microenvironment that inhibits migrating axons and interferes with functional recovery in the long term [57]. Given the complex functional role of reactive astrocytes, it is unclear whether the finding of glial scar reduction after ep-iPSC-NPC transplantation was beneficial or detrimental for functional recovery in this study. Previous studies have reported that transplanted NPCs reduce glial scar formation and functional recovery as found in our study [11, 43, 58–60]. One possible explanation is that a reduction in glial scarring is attributable to the indirect effect of transplanted ep-iPSC-NPCs by the amelioration of inflammation. Astrocytes are rapidly activated by proinflammatory cells, such as microglia and macrophages, and form a glial scar when inflammation is prolonged and extensive following brain ischemia [57]. As transplanted NPCs effectively regulate microglial activation and change their characteristics toward an anti-inflammatory phenotype, glial scar formation might be less extensive in rats transplanted with NPCs than those that did not receive NPCs.

Some transplanted cells themselves differentiated into astrocytes; it is unknown how astrocytes derived from transplanted ep-iPSC-NPCs play a hazardous or beneficial role in functional recovery. It is conceivable that there was little effect on functional recovery in this study as the astrocytes derived from ep-iPSC-NPCs were extremely few in number. Further research is needed to dissect the mechanism of NPC transplantation on reactive astrogliosis in the ischemic brain and its contributory role in functional recovery.

## 5. Conclusions

In the present study, transplantation of ep-iPSC-NPCs induces a long-term functional recovery in a rodent stroke model. The generation of clinical-grade iPSC-NPCs in integration- and xeno-free conditions is critically important for clinical translation in the future. Grafted ep-iPSC-NPCs differentiated into neuronal lineages and formed a functional network with host neurons. Additionally, grafted ep-iPSC-NPCs enhanced endogenous brain repair, including SVZ neurogenesis, and reduced poststroke inflammation. Such effects of ep-iPSC-NPCs ameliorate the microenvironment of the stroke-damaged brain and are responsible for long-term functional recovery. Although much work is required before clinical translation can be considered, our results provide strong evidence that the multimodal therapeutic actions of clinical-grade ep-iPSC-NPCs are a promising opportunity for the treatment of patients with stroke.

## Data Availability

The data used to support the findings of this study are available from the corresponding author upon request.

## Supplementary Materials

Supplementary Materials Supplementary Fig. 1: schematic diagram of in vivo experiments. Supplementary Fig. 2: engrafted ep-iPSC-NPC in ischemic brains following intracerebral transplantation. Supplementary Fig. 3: BDNF expression of human ep-iPSC-NPC in vitro and in vivo. Supplementary Fig. 4: immunofluorescent staining of ED1 and Iba-1 in the brain of the ep-iPSC-NPC group, the fibroblast group, and the vehicle group (*n* = 5 in each group). Supplementary Table 1: primary antibody list.
