# Supplementary material for: Multimodal Therapeutic Effects of Neural Precursor Cells Derived from Human-Induced Pluripotent Stem Cells through Episomal Plasmid-Based Reprogramming in a Rodent Model of Ischemic Stroke
Source: Stem Cells Int. 2020 Mar 23;2020:4061516. doi: 10.1155/2020/4061516 (PMC7125504; doi:10.1155/2020/4061516)
Supplement: Supplementary Materials — Supplementary Fig. 1: schematic diagram of in vivo experiments. Supplementary Fig. 2: engrafted ep-iPSC-NPC in ischemic brains following intracerebral transplantation. Supplementary Fig. 3: BDNF expression of human ep-iPSC-NPC in vitro and in vivo. Supplementary Fig. 4: immunofluorescent staining of ED1 and Iba-1 in the brain of the ep-iPSC-NPC group, the fibroblast group, and the vehicle group (n = 5 in each group). Supplementary Table 1: primary antibody list. [file 4061516.f1.doc]

**Supplementary Materials**

Multimodal therapeutic effects of neural precursor cells derived from human-induced pluripotent stem cells through episomal plasmid–based reprogramming in a rodent model of ischemic stroke

Seung-Hun Oh; Yong-Woo Jeong; Wankyu Choi; Jeong-Eun Noh; Suji Lee; Hyun-Sook Kim, and Jihwan Song

**Supplementary Fig. 1.** Schematic diagram of *in-vivo* experiments

**Supplementary Fig. 2.** Engrafted ep-iPSC-NPC in ischemic brains following intracerebral transplantation

**Supplementary Fig. 3.** BDNF expression of human ep-iPSC-NPC in *in-vitro* and *in-vivo*

**Supplementary Fig. 4.** Immunofluorescent staining of ED1 and Iba-1 in the brain of

the ep-iPSC-NPC group, fibroblast group, and vehicle group (n = 5 in each group)

**Supplementary Fig. 1.** Schematic diagram of *in-vivo* experiments. (A) Timetable of induction of rodent stroke model and intracerebral ep-iPSC-NPCs transplantation. MCAo indicates middle cerebral artery occlusion. FG indicates fluorogold. MEP indicates motor evoked potential study. IHC indicates immunohistochemistry. W means week. (B) Location of ep-iPSC-NPC transplantation. The 1×10^6^ ep-iPSC-NPCs were transplanted at the two sites of peri-infarct areas as following coordinate: 1) (AP: +1.6 mm, ML: -2.0 mm, DV: -3.0 mm and 2) AP: +1.6 mm, ML: -2.0 mm, DV: -6.0 mm.

**Supplementary Fig. 2.** Engrafted ep-iPSC-NPC in ischemic brains following intracerebral transplantation. (A) The 3,3’-diaminobenzidine (DAB) immunostaining of human nucleus (hNu) in the ep-iPSC-NPC transplanted brain. The right panel images are the zoom-up images of two sites of injection (a and b) in the left panel image. (B) Double immunofluorescent staining of hNu and Nestin in the ep-iPSC-NPC transplanted brain. The right panel images are the zoom-up images of the left image (c and d). The samples were count-stained with DAPI. Scale bar: 50μm.

**Supplementary Fig. 3.** BDNF expression of human ep-iPSC-NPC in *in-vitro* and *in-vivo*. (A) Double immunofluorescence staining of brain-derived neurotrophic factor (BDNF) in the ep-iPSC–NPC transplanted brain. The human-specific marker (hNu) was used to detect grafted cells. All samples were counter-stained with DAPI. Scale bars: 50 μm. ep-iPSC–NPCs, neural precursor cells differentiated from induced pluripotent stem cells; MCAo, middle cerebral artery occlusion. (B) Western blot of BDNF in human ep-iPSC-NPCs and human dermal fibroblasts in normoxic- and hypoxic conditions. **p* < 0.05 by Mann-Whitney U test.

**Supplementary Fig. 4.** Immunofluorescent staining of ED1 and Iba-1 in the brain of the ep-iPSC-NPC group, fibroblast group, and vehicle group (n = 5 in each group). The samples were count-stained with DAPI. Scale bar: 50μm. Data were shown as mean ± SEM, *: *p* < 0.05 by one-way ANOVA.

Supplementary Table 1. Primary antibody list

| Name | Host | Dilution rate | Manufacturer |
| --- | --- | --- | --- |
| Anti-human nuclei (hNu) | Mouse monoclonal | 1:100 | Chemicon |
| Anti-mitochondria (hMito) | Rabbit polyclonal | 1:250 | Chemicon |
| MTC02 | Rabbit polyclonal | 1:200 | Novus |
| Anti-Nestin | Rabbit polyclonal | 1:200 | Covance |
| Anti-microtubule associated protein-2 (MAP2) | Rabbit polyclonal | 1:200 | Chemicon |
| Anti-Glutamate decarboxylase 65&67 | Rabbit polyclonal | 1:250 | Chemicon |
| Anti-Darpp32 | Rabbit polyclonal | 1:100 | Cell signaling |
| Anti-GABA | Rabbit polyclonal | 1:1000 | Pel-Freeze |
| Anti-O4 | Mouse monoclonal | 1:250 | Chemicon |
| Anti-GFAP | Mouse monoclonal | 1:500 | Bioscience |
| Anti-BDNF | Rabbit polyclonal | 1:200 | Miilipore |
| Anti-BrdU | Mouse monoclonal | 1:250 | BD Pharmingen |
| Anti-Doublecortin (DCX) | Rabbit polyclonal | 1:200 | Cell signaling |
| Anti-Proliferating cell nuclear antigen (PCNA) | Rabbit polyclonal | 1:250 | Santacruz |
| Anti-Polysialic acid-NCAM (PSA-NCAM) | Mouse monoclonal | 1:250 | Chemicon |
| Anti-ED-1 | Mouse monoclonal | 1:250 | Milipore |
| Anti-Iba-1 | Rabbit polyclonal | 1:250 | Wako |
| Anti-iNOS | Mouse monoclonal | 1:200 | Santacruz |
| Anti-CD206 | Goat polyclonal | 1:200 | Santacruz |
| Anti-caspase3 | Rabbit polyclonal | 1:200 | Milipore |
